# Integrated Metabolomic and Transcriptomic Analysis of *Nitraria* Berries Indicate the Role of Flavonoids in Adaptation to High Altitude

**DOI:** 10.3390/metabo14110591

**Published:** 2024-11-01

**Authors:** Qing Zhao, Jie Zhang, Yanhong Li, Zufan Yang, Qian Wang, Qiangqiang Jia

**Affiliations:** 1 State Key Laboratory of Plateau Ecology and Agriculture, Qinghai University, Xining 810016, China; 18055258641@163.com (Q.Z.); 18309347283@163.com (Y.L.); yang9802192024@163.com (Z.Y.); wq39km@163.com (Q.W.); 2 Department of Pharmacy, Medical College, Qinghai University, Xining 810016, China; 3 Department of Basic Medicine, Qinghai Institude of Health Sciences, Xining 810000, China; jia110829@163.com

**Keywords:** *Nitraria* berries, metabolomic, transcriptomic, flavonoids, altitude

## Abstract

**Background:** Plants of *Nitraria*, belonging to the Zygophyllaceae family, are not only widely distributed at an altitude of about 1000 m but also at an altitude of about 3000 m, which is a rare phenomenon. However, little is known about the effect of altitude on the accumulation of metabolites in plants of *Nitraria*. Furthermore, the mechanism of the high–altitude adaptation of *Nitraria* has yet to be fully elucidated. **Methods:** In this study, metabolomics and transcriptomics were used to investigate the differential accumulation of metabolites of *Nitraria* berries and the regulatory mechanisms in different altitudes. **Results:** As a result, the biosynthesis of flavonoids is the most significant metabolic pathway in the process of adaptation to high altitude, and 5 Cyanidins, 1 Pelargonidin, 3 Petunidins, 1 Peonidin, and 4 Delphinidins are highly accumulated in high–altitude *Nitraria*. The results of transcriptomics showed that the structural genes *C*4*H* (2), *F*3*H*, 4*CL* (2), *DFR* (2), *UFGT* (2), and *FLS* (2) were highly expressed in high–altitude *Nitraria*. A network metabolism map of flavonoids was constructed, and the accumulation of differential metabolites and the expression of structural genes were analyzed for correlation. **Conclusions:** In summary, this study preliminarily offers a new understanding of metabolic differences and regulation mechanisms in plants of *Nitraria* from different altitudes.

## 1. Introduction

Tibetan Plateau, known as the “third pole” of the earth, is the highest plateau in the world, with an average altitude of more than 4000 m [1,2]. Due to its special geographical location and natural conditions, the Tibetan Plateau has various unique berry resources [3]. These plants face a variety of abiotic stresses in the high–altitude environment, such as low oxygen, low temperature, and intense ultraviolet radiation [4]. To adapt to such an extreme environment, high–altitude plants have developed many unique and highly active chemical components. In turn, these components play an essential role in adapting plants to a high–altitude environment and have significant potential for development and exploitation.

As medicinal plants grow and develop in the field, medicinal plants are faced with various environmental threats [4,5]; plants have evolved a diverse array of protective mechanisms that lead to metabolic changes, transcriptional factors (TFs), and genes [6,7,8]. Therefore, studying metabolites and TFs, the genes of plants growing in natural habitats under different environmental variables, is crucial for a broader understanding of metabolite biosynthesis.

However, research about how metabolic constituents of medical plants respond to changes in altitude is limited. Consequently, multi–omics analysis is required to uncover the underlying molecular pathway of metabolite biosynthesis in medicinal plants found at high altitudes. Metabolomics aims to study the changes in the types and amounts of metabolites and their patterns in organisms due to genetic alterations or external environmental stimulation [9]. Dong et al. analyzed metabolite accumulation in *Rhodiola crenulata* and *Rhodiola rosea* plants collected from two altitudes, 2907 m, and 5116 m, using a UPLC–QqQ–MS–based metabolomics approach. Flavonoids featuring glucosides and phenolic components in the two species are positively correlated with altitudes [10]. Transcriptome sequencing (RNA–Seq) is a high–throughput, high–sensitivity, high–resolution technology for analyzing functional genes and regulatory mechanisms in medicinal plants. The most significantly and differentially expressed top 50 genes of the *Potentilla bifurca* in the high–altitude *Potentilla bifurca* were derived from plants that responded to abiotic stress, such as peroxidase, superoxide dismutase protein, and the ubiquitin–conjugating enzyme [11]. Small molecule metabolites are the connecting bridge between genes and phenotypes, and the analysis of metabolomics is more capable of directly reflecting the physiological state of organisms, thus revealing biological mechanisms more precisely. The joint analysis of transcriptomics and metabolomics, widely used in plant metabolism studies, can better find the key metabolic pathways, key regulatory genes, and molecular mechanisms to explain the physiological states and responses of plants. Liu et al. integrated metabolomic and transcriptomic studies to evaluate how cultivation at three altitudes (800 m), (1800 m), and (3600 m) affects flavonoid biosynthesis in pigmented potato tubers. Both red and purple potato tubers grown at high altitudes contain the highest flavonoid content, followed by those grown at low altitudes. Co–expression network analysis revealed three modules containing genes that were positively correlated with altitude–responsive flavonoid accumulation [12].

Plants of *Nitraria* belong to the Zygophyllaceae family; the berries of *Nitraria*, not only have abundant nutritional components but are also rich in bioactive components, such as amino acids, alkaloids, flavonoids, anthocyanins [13,14,15,16]. The *Nitraria* berries have a long history of medicinal use and show high medicinal value. *Nitraria tangutorum* Bor. and *Nitraria sibirica* Pall. have been paid more attention. Most plants grow only in similar or similar climatic environments [17], but plants of *Nitraria* are widely distributed in Shaanxi, Gansu, and Inner Mongolia at an altitude of about 1000 m. In addition, plants of *Nitraria* are also widely distributed on the Tibetan Plateau at an altitude of about 3000 m [18]. This phenomenon is infrequent. Our team’s preliminary research found that *Nitraria* berries are rich in anthocyanins and flavonoids [19]. These components are closely related to resistance (drought, cold, chilling, ultraviolet (UV) radiation) [20,21], which may be an essential material basis for the ability of *Nitraria* plants to adapt to a high–altitude environment. However, studies addressing this scientific issue have yet to be reported. This project was based on the comprehensive analysis of the metabolites and genes of *Nitraria* berries from altitudes of 3548 m and 1500 m. The UHPLC–QE–MS technical platform was applied to obtain the metabolic profiles of *Nitraria* berries. The transcriptomics technology was used to explore the regulation mechanism of the main differential accumulation metabolites. Through the combined analysis of transcriptomics and metabolomics, key metabolic pathways and genes were found to explain the metabolic differences between the adaptation mechanism of *Nitraria* plants to the high–altitude environment. This paper aims to provide a deep understanding of the mechanisms of *Nitraria* plants and their adaptation to high–altitude environments.

## 2. Materials and Methods

### 2.1. Plant Material

*Nitraria* tangutorum Bor: the leaves are hairy, and the berries are hairless. *Nitraria* sibirica Pall: the berries are small and hairless, and the plants of NT and NS are upright. The completely mature berries are dark red. Berries of *Nitraria tangutorum* Bor. (HNT) and *Nitraria sibirica* Pall. (HNS) located at a high altitude were collected in Wulan County, Qinghai Province (36°58′ E, 98°16′ N, altitude of 3548 m). Berries of *Nitraria tangutorum* Bor. (LNT), *Nitraria sibirica* Pall. (LNS) located at a low altitude were collected in Shandan County, Gansu Province (38°58′ E, 101°10′ N, altitude of 1500 m). To avoid the sampling bias, the sampling time of the two locations was 10:00 a.m. using the same sampling method. The ripeness of HNT, LNT, HNS, and LNS berries was evaluated through their apparent color and shape by Doctor Ren Fei of Qinghai University. All the *Nitraria* berries were collected from the sandy environment. We mainly identified two species of *Nitraria* based on the leaves and vegetation.

All the berries were immediately frozen in liquid nitrogen and stored at −80 °C. A total of four groups of samples were collected, with six samples in each group for metabolomics analysis, and then six samples were randomly mixed into three repeats equally for transcriptomic determination. The paired comparison groups were as follows: HNT vs. LNT, HNS vs. LNS.

### 2.2. Chemicals and Reagents

LC–MS–grade acetonitrile and methanol were attained from CNW Technologies (Duesseldorf, Germany). 2–Chloro–L–phenylalanine (internal standard in LC–MS analysis) was purchased from Hengbai Biotechnology Co., Ltd. (Shanghai, China). Six types of standards were used: Kaempferol–3–O–rutinoside, Rutin, Epicatechin, Chlorogenic acid, Quercetin, and Kaempferol, purchased from Sigma Aldrich Co. (St. Louis, MO, USA). The purity of all standard products was greater than or equal to 97%.

### 2.3. Metabolomic Analysis

#### 2.3.1. Samples Preparation

A total of 50 g of *Nitraria* berries was frozen and ground in liquid nitrogen. The sample was ultrasonically extracted with 0.1% hydrochloric acid in 80% ethanol (solid/liquid ratio of 1:5 g/mL) for 30 min and was repeated three times; the mixture was filtered and centrifuged at 12,000 rpm and 4 °C for 15 min. The resulting supernatants were transferred to LCMS vials and stored at −80 °C until UHPLCOrbitrap MS analysis. The quality control (QC) samples were prepared by mixing an equal aliquot of the supernatants from all the samples.

#### 2.3.2. UHPLC–MS for Qualitative Analysis

LCMS/MS analysis was performed using a UHPLC 1290 system (Agilent Technologies, Santa Clara, CA, USA) with a UPLC HSS T3 column (2.1 mm × 100 mm, 1.8 μm) coupled to a Q Exactive Orbitrap MS (Thermo, Waltham, MA, USA). The mobile phase A was 0.1% formic acid in water for positive mode, and the mobile phase B was methanol. The elution gradient was set as follows: 0 min, 1% B; 10 min, 1% B; 25 min, 10% B; 30 min, 10% B; 40 min, 15% B; 60 min, 20% B; 70 min, 20% B; 90 min, 25% B; and 110 min, 100% B. The flow rate was 0.4 mL/min. The injection volume was 2 μL.

ESI was simultaneously used in the positive and negative modes in which the full MS–ddMs^2^ scan mode was operated for analysis. The full MSddMS^2^ scan ranged from 100 to 1500 m/z. ESI source conditions were set as follows: sheath gas flow rate at 13.5 L/min; aux gas flow rate at 4.5 L/min; capillary temperature at 320 °C; full MS resolution as 70,000; MS/MS resolution as 17,500; collision energy as 20/40/60 eV in the NCE model; and spray voltage as 3.8 kV (positive mode) or 3.1 kV (negative mode), respectively. The full MS–ddMS^2^ scan speed was 12 Hz/ms.

#### 2.3.3. UHPLC–MS Quantitative Analysis

Chromatographic conditions for quantitative analysis as Section 2.3.2. The mobile phase A was 0.9% acetic acid in water, and B was methanol. The elution gradient was set as follows: 0 min, 20% B; 9 min, 100% B; 10 min, 100% B; 12 min, 20% B; and 14 min, 20% B. Mass spectrum conditions refer to quantitative analysis as Section 2.3.2.

#### 2.3.4. Establishment of the In–House Database for *Nitraria* Berries

To ensure the reliability of peak identification, an in–house database of *Nitraria* berries was established by integrating the TCMSP (traditional Chinese medicine system pharmacology database, https://old.tcmsp-e.com/tcmsp.php (accessed on 15 July 2023)), TCMID (traditional Chinese medicine integrated database, http://www.megabionet.org/tcmid (accessed on 15 July 2023)), ChemSpider (http://www.chemspider.com/ (accessed on 16 July 2023)), Chemical Book (http://www.chemicalbook.com/ (accessed on 16 July 2023)) and related studies. Finally, the formulas of 1000 known compounds were collected and listed in an Excel spreadsheet, including the compound name, molecular formula, molecular weight, structure diagram, and natural source.

#### 2.3.5. Procedure for the Identification of Unknown Constituents

We imported the collected primary mass spectrometry raw data into Thermo Scientific Compound Discoverer 3.0 software through automatic screening for the established in–house database, the compounds of which matched the determined molecular formula and were found as potential candidates. A list of compounds with matching scores higher than 85.00 was created manually, including the information on accurate precursor ion masses and retention times. Based on this list, targeted MS/MS detection was carried out. Candidate compounds were identified by their retention times, accurate masses within an error of 5 ppm, and reasonable fragmentation pathways, confirmed by available standards. Moreover, using the online database Mzcloud (https://www.mzcloud.org/ (accessed on 20 July 2023)) further supplements and validates the search results of the self–built database.

### 2.4. Transcriptome Analysis

#### 2.4.1. RNA Extraction, Library Construction and Sequencing

The extraction of total RNA was performed with three biological replicates from each group. The total RNA of *Nitraria* berries was extracted using the Trizol reagent kit according to the manufacturer’s protocol. The RNA purity was checked using the NanoPhotometer spectrophotometer (IMPLEN, Dortmund, Germany). A Qubit RNA Assay Kit in a Qubit 2.0 Fluorometer (Life Technologies, Carlsbad, CA, USA) was used to measure the RNA concentration. The RNA integrity was assessed on an Agilent 2100 Bioanalyzer (Agilent, San Jose, CA, USA). The degradation and contamination were tested on 1% agarose gel. The construction of the library and the RNA–Seq was performed on Illumina HiSeqTM 4000 by Gene Denovo Biotechnology Co., Ltd. (Panyu, China).

#### 2.4.2. Filtering of Clean Reads

Reads obtained from the sequencing machine included raw reads containing adapters or low–quality bases, which could affect the following assembly and analysis. Thus, to achieve high–quality clean reads, reads were further filtered by fastp [22] v0.18.0. The screening criteria were as follows: (1) removing reads containing adapters; (2) removing reads containing more than 10% of unknown nucleotides; (3) removing low–quality reads containing more than 50% of low–quality (Q–value ≤ 20) bases.

#### 2.4.3. De Novo Assembly and Functional Annotation

The high–quality clean reads were assembled de novo using the Trinity platform [9]. The clean reads were mapped to the reference transcriptome using the short reads alignment tool Bowtie2 with default parameters, and the mapping ratio was calculated. To annotate the unigenes, we used the BLASTx program (http://www.ncbi.nlm.nih.gov/BLAST/ (accessed on 6 August 2023)) with an E–value threshold of 1 × 10^−5^ for the NCBI non–redundant protein (Nr) database (http://www.ncbi.nlm.nih.gov (accessed on 10 August 2023)), the Swiss–Prot protein database (http://www.expasy.ch/sprot (accessed on 12 August 2023)), the Kyoto Encyclopedia of Genes and Genomes (KEGG) database (http://www.genome.jp/kegg (accessed on 29 August 2023)), and the COG/KOG database (http://www.ncbi.nlm.nih.gov/COG (accessed on 29 August 2023))). 

#### 2.4.4. Enrichment Analysis of Differentially Expressed Genes

To obtain high–quality clean reads, reads were further filtered by fastp [22] v0.18.0. The identification of differentially expressed genes (DEGs) was performed by DESeq2 [23]. The genes with the parameters of a false discovery rate (FDR) < 0.05 and |log_2_(fold change)| ≥ 1 were considered as DEGs. All the DEGs were mapped to the KEGG pathway database. The formula of the hypergeometric test calculated the significance of DEG enrichment.

### 2.5. Correlation Analysis of the Metabolomic and Transcriptomic

The transcriptomic and metabolomic data show the differences in gene expression and metabolite levels. However, transcription and metabolism do not occur independently in biological systems. To reveal the regulatory mechanism between gene expression and metabolite accumulation, we can analyze their relationship based on the rule that “genes or metabolites involved in the same biological process have the same or similar changes”. To obtain the sets of associated genes and metabolites that influence the sample grouping and analyze the association characteristics, the KEGG pathway model was analyzed based on the data of gene expression and metabolite abundance in each paired comparison group. Pearson correlation coefficients were calculated for differential metabolites and DEGs. The strongly correlated DEGs and differential metabolites were connected and plotted using CytoScape v3.8.0 software based on the calculation results.

### 2.6. Statistical Analysis

The collected raw data were imported into Compound Discoverer 3.0 software for baseline correction, peak calibration, deconvolution analysis, etc. The molecular weight error was set to 5 ppm, and the retention time error was set to ±0.1. The dataset containing the peak number, sample name, and normalized peak area was transferred to SIMCA–P 14.1 (Umetrics, Vasterbottens Lan, Sweden) software. Principle component analysis (PCA) was performed to visualize the distribution and grouping of the samples. The variable importance projection value (VIP) was obtained by orthogonal projection for latent structure discriminate analysis (OPLS–DA) mode analysis. The *p* value between the two sets of data was calculated by a *t*-test, and the compounds with VIP > 1.0 and *p* < 0.05 were used as differential accumulation metabolites. The change level of differential accumulation metabolite expression was reflected by calculating the fold change value. The corresponding metabolic pathways of differentially accumulated metabolites were analyzed by the KEGG database and metabolomic data processing platform MetaboAnalyst (https://www.metaboanalyst.ca (accessed on 2 September 2023)). Using SPSS Statistics 25.0, the statistical significance was calculated through Student’s *t*-test. The significance level was set as *p* < 0.05 (significant) and *p* < 0.01 (extremely significant). Fold change was calculated to reflect the alteration levels of metabolites and gene expression.

## 3. Results

### 3.1. Metabolic Profiling

The filtered and identified results and secondary fragmentation information of all compounds are shown in Table S1. A total of 103 compounds, including 22 anthocyanins, 30 flavonoids, 25 phenolic acids, 9 organic acids, 13 amino acids, and 4 sugars, were preliminarily identified by the retention time, precise molecular weight, and MS/MS fragment ion comparison.

**Figure 1:**
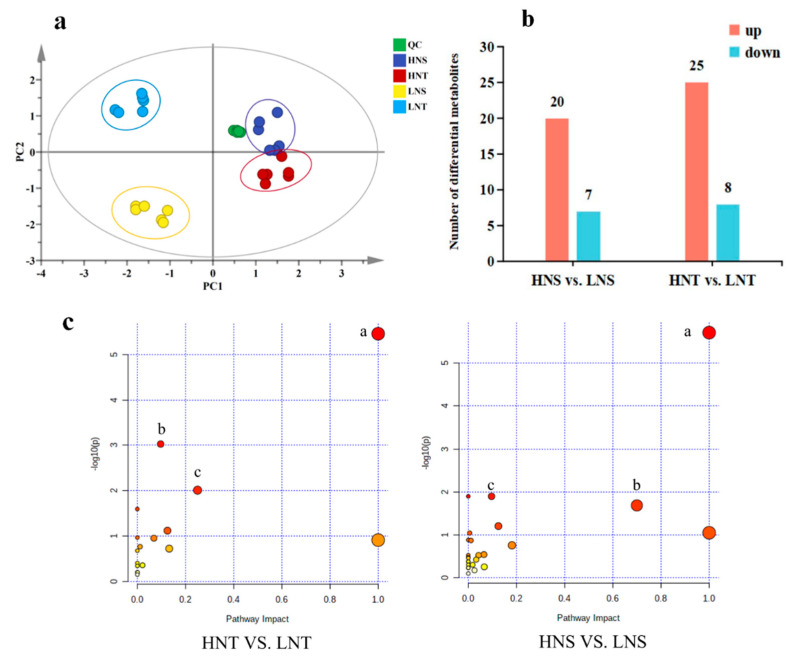
The PCA score plot of metabolites in HNT, LNT, HNS, and LNS (**a**). The amount of up−regulated and down–regulated DAMs; (**b**) the results of DAM pathway analysis of *Nitraria* berries; and (**c**) the results of DAM pathway analysis of *Nitraria* berries at different altitudes. The color from yellow to red indicates that the smaller the *p* value, the larger the diameter of the circle, indicating that the number of metabolites enriched in the pathway is more. (a: Anthocyanin biosynthesis; b: Flavone and flavonol biosynthesis; c: Valine; leucine and isoleucine biosynthesis).

To reveal the effects of different altitudes on the metabolism of *Nitraria* berries, utilizing non–targeted metabolomics technology, we investigated the metabolic alterations between different altitudes. The PCA score results (Figure 1a) showed that out of HNS, HNS, and LNT, HNT were clearly separated both in the positive and negative modes. By observing the dispersion of quality control samples, the stability and reliability of instrumental analysis could be guaranteed. The OPLS–DA and Student’s *t*-test were performed for each paired comparison group to obtain VIP and *p* values, respectively. The metabolites with VIP > 1.0 and *p* < 0.05 were considered differential metabolites (DAMs). These DAMs were further identified through tandem mass spectrometry and annotated using an in–house MS/MS database. The number of differentially accumulated metabolites that were up–regulated and down–regulated in each comparison group is shown in Figure 1b and Table S1. For HNT vs. LNT, a total of 33 differentially accumulated metabolites were detected in the control group, of which 25 were up–regulated and 8 were down–regulated; for HNS vs. LNS, a total of 27 differentially accumulated metabolites were detected in the control group, of which 20 were up–regulated, and 7 were down–regulated. There were 24 common DAMs in the two high– and low–altitude comparison groups. Cyanidin–3–O–diGlucoside, Delphinidin–glucuronide, Delphinidin–dihexoside (II) a, Pelargonidin–3–Glu/isomer, Kaempferol–3–O–rhamnosylgalactoside–7–O–glucoside, p–Coumaric acid, and Rutin were significantly up–regulated in the two high– and low–altitude contrast groups. The down–regulated differential metabolites included Valine, Tyrosine, 3–Isopropylmalate, and 2–Ketobutyric acid. The results of differential accumulation metabolites included the comparison of different altitudes (VIP, P, FC), as shown in Table S2. The differentially accumulated metabolites mainly include anthocyanins, flavonoids, and amino acids.

### 3.2. Pathway Enrichment of DAMs

Using KEGG enrichment analysis, we determined that all the DAMs in positive and negative modes were involved in many metabolic pathways. The pathways with an impact value > 0.1 and statistical significance (*p* < 0.05) were selected as potential differential metabolic pathways in Figure 1c. The two paired comparison groups with altitudes had four significantly identical pathways. From the enrichment results of DAMs, it was evident that anthocyanin biosynthesis, flavonoids, and flavonol biosynthesis are important metabolic pathways co–existing in HNS vs. LNS and HNT vs. LNT.

**Figure 2:**
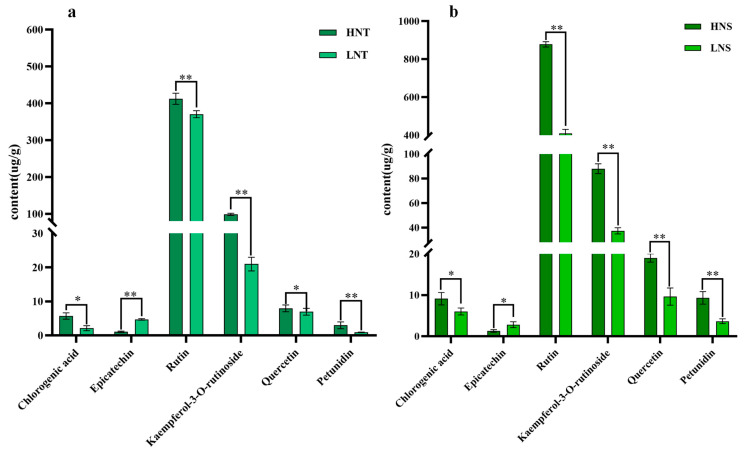
Content of 6 metabolites in NT and NS at different altitudes (* *p* < 0.05, ** *p* < 0.01): (**a**) HNT vs. LNT; (**b**) HNS vs. LNS.

To further validate the results of metabolomics analysis, we selected six different metabolites for validation and the comparative analysis of six different metabolites in NT and NS at different altitudes. The quantitative results shown in Figure 2 were consistent with the trend of screening differential metabolites.

### 3.3. Transcriptome Sequencing, Assembly, and Annotation

**Figure 3:**
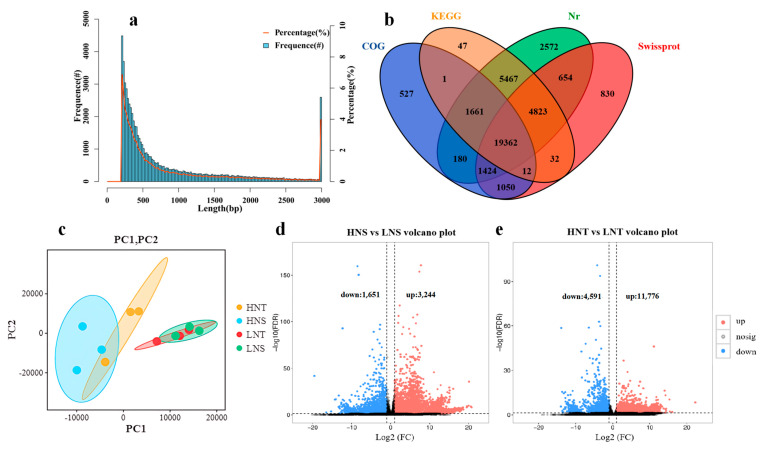
The length distribution of de novo assembled unigenes: (**a**) Venn diagram of unigene annotation results from four databases; (**b**) Principal component analysis; and (**c**) volcano plots to filter the DEGs of berries in different altitudes groups of HNS vs. LNS (**d**) and HNT vs. LNT (**e**).

After sequencing the cDNA libraries, the number of clean reads per library ranged from 42,900,204 to 61,170,750 after filtering out the low–quality data (Table S3). A total of 65,398 unigenes were obtained from the libraries of the four different groups, with 43.61% having a GC content and 17.12% an N50 content, an N50 of 1535 bp, and an average length of 874 bp (Table S3). There were 26,870 (41.09%) unigenes of 200–400 bp; 11,235 (17.18%) unigenes of 400–600 bp; 5757 (8.80%) unigenes of 600–800 bp; 3761 (5.75%) unigenes of 800–1000 bp; 2889 (4.42%) unigenes of 1000–1200 bp; 2323 (3.55%) unigenes of 1200–1400 bp; 1996 (3.05%) unigenes of 1400–1600 bp; 1844 (2.82%) unigenes of 1600–1800 bp; 1590 (2.43%) unigenes of 1800–2000 bp; and 7133 (10.91%) unigenes were > 2000 bp (Figure 3a).

The annotation results of all unigenes are shown in Table S4. As is shown, 38,642 (59.09%) out of 65,398 unigenes were annotated. In total, 31,405 (48.02%), 24,217 (37.03%), 28,187 (43.10%), and 36,143 (55.27%) were annotated in the databases of KEGG, COG, SwissProt, and Nr, respectively. The Venn plot (Figure 3b) of the unigene annotation results shows that 19,362 unigenes (50.11% of annotated unigenes) exist in all four databases, and only 3976 unigenes (10.29% of annotated unigenes) are included in the single database.

### 3.4. Comparison of Gene Expression

We used transcriptomic technology to study the changes in genes in the four groups. as mentioned in the PCA score results. Figure 3a shows that HNS, HNT, LNS, and LNT were clearly separated. Differentially expressed genes (DEGs) in all the paired comparison groups were identified using DESeq2, with FDR < 0.05 and |log_2_(fold change)| > 1. They were found in HNS vs. LNS 4,895 DEGs (3244 up–regulated and 1651 down–regulated) and 16,367 DEGs (11,776 up–regulated and 4591 down–regulated) in HNT vs. LNT (Figure 3b,c).

### 3.5. KEGG Pathway Enrichment Analysis of DEGs

In order to further determine the biological metabolic pathways and functions of DEGs in *Nitraria* berries at different altitudes, the KEGG pathway classification and enrichment analysis of DEGs were carried out in this study. The results showed that the primary metabolic pathways of DEGs annotated by *Nitraria* berries in different altitude comparison groups were similar, belonging to five categories. Among them, the Metabolism category contained the largest number of differentially expressed genes and contained the largest number of secondary pathways. In order to further screen the related regulatory genes of DAMs, this study further analyzed the secondary pathways under the category of metabolism. This category includes secondary pathways such as carbohydrate metabolism, amino acid metabolism, lipid metabolism, the metabolism of cofactors and vitamins, and the biosynthesis of other secondary metabolites. The DEGs involved in the biosynthesis pathways of amino acid metabolism, lipid metabolism, and other metabolites are closely related to the regulation of the main differential accumulation metabolites screened in the third chapter.

**Figure 4:**
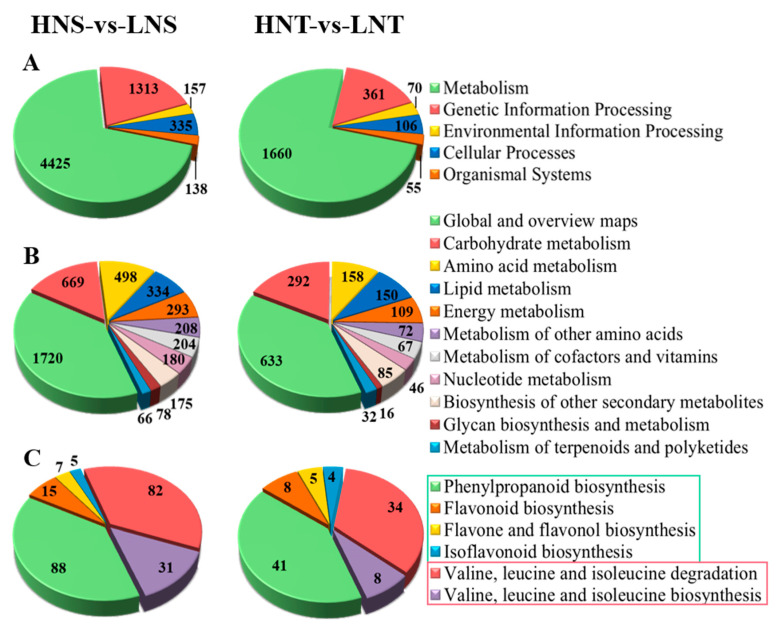
KEGG enrichment analysis and functional classification of DEGs in different altitudes groups: (**A**) KEGG primary classification results of DEGs; (**B**) secondary classification results of DEGs; and (**C**) tertiary classification results of DEGs enriched into pathways.

For the comparison groups of two types of *Nitraria* berries (NT and NS) at different altitudes, the differentially accumulated metabolites were mainly enriched in flavonoids (including anthocyanin) and amino acid metabolic pathways, involving two secondary pathways: the biosynthesis of other metabolites and amino acid metabolism. Therefore, this study further analyzed the tertiary pathways under these two secondary pathways and found that DEGs were mainly enriched in tertiary pathways such as phenylpropanoid biosynthesis, flavonoid biosynthesis, isoflavonoid biosynthesis, the biosynthesis of flavones and flavonols, and the biosynthesis of Valine, leucine, and isoleucine. The number of DEGs in each pathway is shown in Figure 4.

### 3.6. Joint Analysis of Metabolomics and Transcriptomics

Based on research on the chemical composition of Nitraria berries, this study utilized non–targeted metabolomics to comprehensively screen for differentially accumulated metabolites in Nitraria berries at different altitudes, and the results showed that flavonoids (including anthocyanins) were the most significantly differentially accumulated metabolites, including Cyanidin, Pelargonidin, Delphinidin, and Petunidin alongside anthocyanin derivatives, flavonoids and their derivatives such as Quercetin and Kaempferol. Through the results of transcriptomic differential expression gene screening, combined with KEGG functional classification and the enrichment analysis of DEGs, several genes involved in encoding key enzymes for flavonoid (including anthocyanins) synthesis, such as CHS, F3H, DFR, ANS, and FLS, were screened out. On this basis, we constructed a metabolic network diagram of flavonoids and their synthesized protein structural genes in Nitraria berries. We analyzed the accumulation of major differentially accumulated metabolites and the expression of structural genes. Phenprobamate is the initial compound in a series of biochemical reactions that lead to the formation of flavonoids in *Nitria* berries.

**Figure 5:**
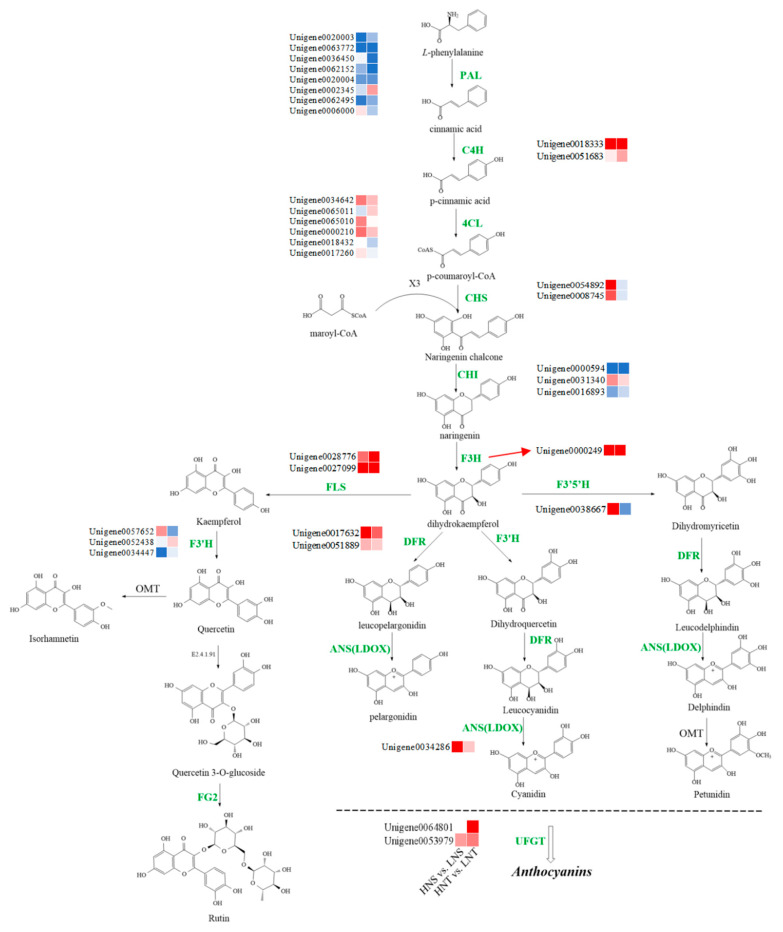
Flavonoid biosynthetic structural genes of DEGs in different altitudes groups. The log_10_FC–transformed values of DEGs are indicated from blue to red (low to high). PAL: phenylalaninammo–ialyase; C4H: cinnamic acid–4–hydroxylase; 4CL: 4–Coumaric acid coenzyme ligase; CHS: chalcone synthase; CHI: chalcone isomerase; F3H: flavanone–3–hydroxylase; FLS: flavonol Synthase; F’3′5′H: flavonoid 3′; 5′–hydroxylase; FLS: flavonol Synthase; DFR: dihydroflavonol 4–reductase; F3′H: fla–vonoid 3′–monooxygenase; ANS: anthocyanidin synthase; FG2: flavonol–3–O–glucoside L–rhamnosyltransferase; UFGT: UDP–glycose flavonoid glycosyltransferase.

Phenylalanine (PAL) deaminase is the first key enzyme in the flavonoid biosynthesis pathway, and in the presence of PAL, PAL is catalyzed by deamidation to trans–cinnamic acid. Cinnamic acid is converted to p–Coumaric acid and the p–Coumaroyl coenzyme by the catalysis of cinnamic acid 4–hydroxylase (C4H) and 4–Coumaric acid coenzyme ligase (4CL), respectively. The condensation of three molecules of malonyl coenzyme and one molecule of the p–Coumaroyl coenzyme to form naringenin chalcone was catalyzed by chalcone synthase (CHS). Subsequently, the naringin chalcone was rapidly converted to naringin by chalcone isomerase (CHI). Naringin is a precursor of flavonols, anthocyanins, proanthocyanidins, flavonoids, and isoflavones. During flavonoid biosynthesis, it is hydroxylated to dihydrokaempferol by naringin 3–dioxygenase (F3H), and then a double bond is introduced to form Kaempferol by flavanol synthase (FLS). Kaempferol is transformed into Quercetin by flavonoid 3′–monooxygenase (F3′H). Quercetin then becomes Quercetin–3–O–glucoside through glucosyltransferase (E2.4.1.91), which is finally converted to Rutin by flavonol–3–O–glucoside L–rhamnosyltransferase (FG2). Dihydrokaempferol is converted to dihydromyricetin by flavonoid 3′,5′–hydroxylase (F3′5′H), which is then turned into Leucodelphindin by dihydroflavanone reductase (DFR). Leucodelphindin is transformed into Delphinidin by the anthocyanin synthesis enzyme (ANS), which then undergoes methylation to become Petunidin. Subsequently, Petunidin is converted into its glycosides by UDP–glycose flavonoid glycosyltransferase (UFGT) and other enzymes. Dihydrokaempferol is converted to Taxifolin by F3′H enzymes, which is then transformed into Leucodelphindin by DFR. Finally, Leucodelphindin becomes Cyanidin under the influence of ANS. Cyanidin is converted into its glycosides by glycosyltransferases like UFGT. The results of metabolomics analysis showed that most of the flavonoids, especially anthocyanins, were significantly accumulated in the Nitraria berries during high–altitude acclimatization. Flavonoids are a kind of important metabolite in plants, and metabolomics analyses showed that most of the flavonoids, especially anthocyanins, were significantly accumulated in Nitraria berries during high–altitude adaptation. The metabolic pathway of these flavonoids and genes is shown in Figure 5 based on the KEGG pathway database. Combining the DEG results and the Pearson correlation analysis, the filtered DEGs and their correlations to these flavonoid compounds are presented. In HNS vs. LNS and HNT vs. LNT, genes encoding key enzymes of the flavonoid biosynthetic pathway, include 2 C4H (Unigene0018333, Unigene0051683), 2 (4CL: Unigene0034642, Unigene0000210), (F3H: Unigene0000249), 2 (FLS: Unigene0028776, Unigene0027099), 2 (DFR: Unigene0017632, Unigene0051889), (ANS: Unigene0034286), and 2 (UFGT: Unigene0064801, Unigene0053979), which were upregulated significantly and consistent with flavonoid accumulation. In addition, the expression of several structural genes encoding alanine deaminase (PAL) showed an opposite trend to flavonoid accumulation, suggesting that PAL may not be playing a rate–limiting role.

### 3.7. Transcription Factors Analysis

**Figure 6:**
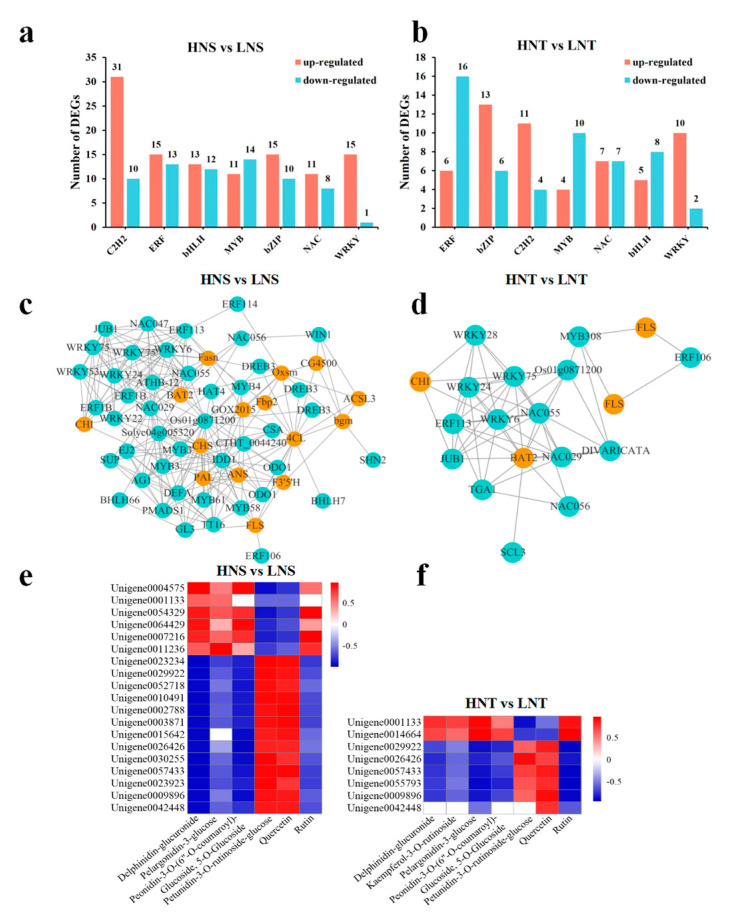
The number and species of differentially expressed transcription factors in HNS vs. LNS (**a**) and HNT vs. LNT (**b**). (**c**,**d**) Protein–Protein interaction analysis of regulatory factors with structural genes in HNS vs. LNS and in HNT vs. LNT; (**e**) correlation analysis of flavonoids and related transcription factors in HNS vs. LNS and (**f**) in HNT vs. LNT.

As a molecular switch, transcription factor (TF) controls various growth and development processes under different environmental stresses. Figure 6a,b illustrates the types and quantities of TFs across the comparison groups of NS and NT at different altitudes. Comparison at high and low altitudes revealed that the types of TFs were significantly differentially expressed in the two species of *Nitraria* berries and were essentially the same, which mainly involved the top seven (sorted by the number of differentially expressed genes) transcription factor families, including C2H2, ERF, bHLH, MYB, bZIP, NAC, and WRKY.

Studies have shown that TFs play an important regulatory role in the biosynthesis of plant flavonoids (anthocyanins). In this study, the differentially expressed TFs in the comparison group of *Nitraria* berries at different altitudes were comprehensively screened. The TFs with the largest number of differentially expressed genes were mainly concentrated in flavonoids (including anthocyanins), such as MYB, bHLH, bZIP, and WRKY. Although the structural genes in the biosynthetic pathway of flavonoids (including anthocyanins) in *Nitraria* berries are analyzed in detail in Figure 5, its biosynthetic regulatory network needs further improvement. Therefore, we analyzed the protein interaction between the corresponding regulatory factors of flavonoids in *Nitraria* berries and structural genes to further reveal the regulation mechanism of flavonoids in *Nitraria* berries at different altitudes.

The analysis of the interaction between flavonoid synthesis genes and TFs in different attitudes of *Nitraria* berries shows that the number of transcription factors screened from HNS vs. LNS group DEGs was the largest, including 41 C2H2, 25 MYB, 25 bHLH, 25 bZIP, and 16 WRKY transcription factors. The analysis results of the transcription factor and synthetic gene protein–protein interaction (Figure 6c,d) showed that 5 WRKY, 4 MYB, 2 bHLH, 4 NACs, and 4 ERFs TFs are directly or indirectly involved in the regulation of *CHI*, *FLS*, *F*3′5′*H*, *ANS*, 4*CL*, *CHS* and other structural genes. Here, 1 ERF106 regulates *FLS* alone, and bHLH7 regulates *4CL* alone. However, the TFs interact with each other to jointly regulate anthocyanin synthesis structural genes. HNT vs. LNT group DEGs have relatively few differential TFs, including 13 bHLH, 14 MYB, 19 bZIP, and 12 WRKY. The regulatory network is relatively simple, where MYB308 TFs and ERF106 co–regulate FLS synthesis structural genes, and the WRKY*28*, WRKY2, WRKY75, WRKY6, ERF113, and NAC055 transcription factors co–regulate *CHI* synthesis structural genes.

As shown in Figure 6e,f, based on transcription factor expression, combined with the results of flavonoids analysis, in the HNS vs. LNS comparison group, homologs of the transcription factors bHLH, ERF, and MYB were significantly and positively correlated (|r| > 0.8) with most of the flavonoids including Delphinidin, Pelargonidin, Peonidin, and Rutin. The accumulation of Petunidin and Quercetin flavonoids was associated with bHLH (Unigene0004575), ERF (Unigene0001133), MYB (Unigene0054329, Unigene0064429, Unigene0007216, Unigene0011236), NAC (Unigene0003871, Unigene0015642, Unigene0026426, Unigene0030255), and WRKY (Unigene0057433, Unigene0023923, Unigene0009896, Unigene0042448) homolog genes, which were significantly positively correlated (|r| > 0.8). A comparable trend was noted in the HNT vs. LNT comparison group, with the TFs ERF (Unigene0001133) and MYB (Unigene0014664) having a significant positive correlation with most flavonoids including Delphinidin, Pelargonidin, Peonidin, Kaempferol and Rutin; Petunidin and Quercetin analogs similarly correlated with ERF (Unigene0029922), NAC (Unigene0026426), and WRKY (Unigene0057433, Unigene0009896, Unigene0042448).

## 4. Discussion

The high–altitude environment, characterized by various abiotic stressors, such as drought, low temperatures, and high salinity, can trigger the generation of ROS, which can decrease the chlorophyll content and photosynthetic efficiency and inhibit the growth of plants. This presents a formidable challenge for plant survival and development at high altitudes. Reactive oxygen species (ROS) are byproducts of photosynthesis in plants, produced in organelles such as chloroplasts, mitochondria, peroxisomes, and apoplasts. Low–to–moderate levels of ROS are beneficial for maintaining plant growth; however, due to their high reactivity and toxicity, the excessive production of ROS can cause oxidative damage to cell membranes, proteins, RNA, and DNA, ultimately leading to plant death [24,25,26,27]. Therefore, effectively scavenging excess ROS is crucial for sustaining normal plant growth.

From the perspective of metabolomics, most flavonoids, especially anthocyanins, are significantly increased in high–altitude *Nitraria* berries compared with the low–altitude group, which might be an important reason why *Nitraria* berries can adapt to growth in high–altitude environments. Anthocyanins are usually found in vacuoles close to ROS production sites [28]. Under abiotic stress, ROS may diffuse rapidly into membranes, different cellular compartments, and the vacuole [29,30]. Anthocyanins have superb antioxidant properties, which can reduce oxidative stress by eliminating abiotic stress–induced ROS, thus enabling plants to adapt to abiotic stresses, thereby protecting them from growth inhibition and cell death [31,32,33]. The ROS–removing effect of anthocyanins has been demonstrated in anthocyanin–deficient leaves (green leaves) and anthocyanin–containing leaves (red leaves), where green leaves have more endogenous ROS and are more sensitive to abiotic stresses, whereas red leaves are able to scavenge more ROS than green leaves [34,35,36].

The accumulation of metabolites and the expression of related synthetic genes were analyzed in two species of *Nitraria* berries (NT and NS) at different altitudes. In total, 35 structural genes for flavonoid synthesis, encoding 12 key synthetic enzymes, were finally screened from DEGs. The expression patterns of 10 of these structural genes were consistent with the changes in flavonoid content in both NT and NS comparator groups, which included *C4H*, 4*CL*, *F*3*H*, *FLS*, *DFR,* and *ANS*; in particular, *C*4*H*, *F*3*H* as well as *FLS* were significantly highly expressed in both groups of high–elevation *Nitraria* berries. It is hypothesized that these genes play a crucial role in regulating flavonoid synthesis during the high–altitude acclimatization of *Nitraria* berries, and their significant high expression contributes to the accumulation of flavonoids. In addition, although *PAL*, *CHI*, and *F*3*′*5′*H* were involved in flavonoid biosynthesis and showed reduced expression levels in the high–altitude group, which was opposite to the pattern of flavonoid accumulation, it was speculated that these three genes were unlikely to play a rate–limiting role.

Transcription factors such as bHLH, ERF, WRKY, MYB, NAC, and ERF have been reported to be involved in fruit development and ripening, among which bHLH, WRKY, and MYB transcription factors are widely involved in the regulation of flavonoid–related synthetic genes [37,38,39]. In the present study, the comparative analysis of the transcriptome data of *Nitraria* berries at high and low altitudes showed that several homologous genes of transcription factors WRKY and MYB were involved in the regulation of flavonoid synthesis in *Nitraria* berries at different altitudes. The expression of genes encoding the MYB type of transcription factors was strongly correlated with the flavonoid content of Delphinidin, Pelargonidin, Peonidin, and Rutin. The flavonoid content had a strong positive correlation with Delphinidin, Pelargonidin, Peonidin, and Rutin. However, a negative correlation was found with Petunidin and Quercetin types, and the expression of WRKY–type transcription factors correlated with the accumulation of flavonoids in exactly the opposite way to that of the transcription factor MYB. These results imply that regulating flavonoids by transcription factors in the berries of *Nitraria* at different altitudes is a complex process, and the regulation of different types of flavonoids by the same class of transcription factors varies, which needs to be further studied. In addition, bHLH–like transcription factors are also involved in the regulation of flavonoid metabolism in *Nitraria* berries at different altitudes. It has been reported that MYB transcription factors can regulate flavonoid synthesis either alone or in combination with bHLH transcription factors to form a complex [40]; so, we hypothesize that there are potentially multiple forms of the regulation of flavonoids in *Nitraria* berries using the transcription factor MYB. Flavonoids based on the phenylalanine biosynthesis pathway, as well as anthocyanins, play a crucial role in resisting environmental stimuli [41,42,43], and in this study, we found that the key synthesis genes of the flavonoid biosynthesis pathway with related transcription factors were activated with increasing altitude, enhancing the accumulation of flavonoids, and enabling *Nitraria* berries to adapt to extreme natural conditions.

To reveal the metabolism differences between *Nitraria* berries from different altitudes, respectively, metabolomic and transcriptomic technologies were combined to investigate the key metabolites and genes. Through the joint analysis, the differences in metabolic mechanisms among different altitudes of *Nitraria* berries were revealed. The results of non–targeted metabolomics research indicate that the biosynthesis of anthocyanins is the most significant metabolic pathway among the different altitudes of *Nitraria* berries, where anthocyanin biosynthesis is the most significantly altered during accumulation at a high altitude, and several anthocyanin analogs involved are highly expressed in high–altitude *Nitraria* berries. Based on transcriptomics technology, many differentially expressed genes from different altitudes were obtained, and association analysis was performed on the differentially accumulated metabolites detected by metabolomics. The structural genes *C*4*H,* 4*CL, F*3*H, FLS*, *DFR,* and *ANS* were highly expressed in high–altitude *Nitraria* berries, which is consistent with the trend of metabolite accumulation, especially for the three structural genes, *C*4*H*, *F*3*H*, and *FLS*, which showed the most significant ploidy changes. In summary, this study, for the first time, conducted a comprehensive comparative analysis of medicinal *Nitraria* berries from different altitudes of transcriptome and metabolome and provides important theoretical support to understand the differences in the chemical components of different altitudes of *Nitraria* berries and to explain the metabolic differences found for the adaptation mechanism of *Nitraria* to high–altitude environments.

## Data Availability

The data that support the findings of this study are available from the corresponding authors upon reasonable request.

## Supplementary Materials

The following supporting information can be downloaded at: https://www.mdpi.com/article/10.3390/metabo14110591/s1. The names of the repository/repositories and accession number(s) can be found below: Genome Sequence Archive: CRA004821. Table S1. Compounds identified in berries of Nitraria (QC) by UHPLC–QE–Orbitrap–MS; Table S2. Some of key metabolites differentially accumulated between LNS vs HNS and LNT vs HNT; Table S3. The number of clean reads per library ranged from 42,900,204 to 611,70,750; Table S4. Gene annotation in four databases. Refs. [18,44,45,46,47,48,49,50,51] are cited in the Supplementary Materials.
