# Supplementary material for: Integrated Metabolomic and Transcriptomic Analysis of Nitraria Berries Indicate the Role of Flavonoids in Adaptation to High Altitude
Source: Metabolites. 2024 Nov 1;14(11):591. doi: 10.3390/metabo14110591 (PMC11596137; doi:10.3390/metabo14110591)
Supplement: Supplementary file 1 [file metabolites-14-00591-s001.zip › metabolites-3239328-supplementary.pdf]

Integrated Metabolomic and Transcriptomic Analysis of *Nitraria* Berries Indicate the Role of Flavonoids in Adaptation to High Altitude

Qing Zhao [1,2,†], Jie Zhang [3,†], Yanhong Li [1,2], Zufan Yang [1,2], Qian Wang [1,2] and Qiangqiang Jia [1,2,*]

[1] State Key Laboratory of Plateau Ecology and Agriculture, Qinghai University, Xining 810016, China; 18055258641@163.com (Q.Z.); 18309347283@163.com (Y.L.); yang9802192024@163.com (Z.Y.); wq39km@163.com (Q.W.)
[2] Department of Pharmacy, Medical College, Qinghai University, Xining 810016, China
[3] Department of Basic Medicine, Qinghai Institude of Health Sciences, Xining 810000, China; jia110829@163.com
* Correspondence: 2015990037@qhu.edu.cn
† These authors contributed equally to this work.

Table S1. Compounds identified in berries of *Nitraria* (QC) by UHPLC-QE-Orbitrap-MS.

No.	Compound	Rt (min)	Formula	Ion	Measured (m/z)	Theoretical (m/z)	Error (ppm)	MS/MS fragment ion [b]	Refs. [d]
					Anthocyanin				
1	Cyanidin-3-O-diglucoside	43.28	$C_{27}H_{31}O_{16}$	$[M]^+$	611.1604	611.1607	-0.49	287.0539$[M-Glu*2]^+$	[1]
2	Delphinidin-dihexoside	44.86	$C_{27}H_{31}O_{17}$	$[M]^+$	627.1564	627.1556	-0.96	319.0436$[M-Glu-Rha]^+$, 481.0898$[M-Rha]^+$, 303.0488$[M-Glu-Rha-OH]^+$	[2]
3	Cyanidin-3-O-(6″-O-coumaroyl)-glucoside	50.49	$C_{27}H_{31}O_{15}$	$[M]^+$	595.1652	595.1656	-0.67	287.0539$[M-Glu-Coumaroyl]^+$	[3]
4	Petunidin 3-O-rutinoside-3-O glucoside	51.21	$C_{34}H_{43}O_{21}$	$[M]^+$	787.2291	787.2265	3.30	641.1731$[M-Rha]^+$, 479.1165$[M-Glu-Rha]^+$, 317.0642$[M-Glu*2-Rha]^+$	[4]
5	Delphinidin-glucoside	51.21	$C_{22}H_{23}O_{12}$	$[M]^+$	479.1190	479.1184	1.26	317.0652$[M-Glu]^+$	[2]
6	Peonidin-3-O-galactoside	53.28	$C_{22}H_{23}O_{11}$	$[M]^+$	463.1238	463.1235	0.07	301.0702$[M-Gal]^+$	[2]
7	Cyanidin-3-O-(cis-6″-O-coumaroyl)-diglucoside	57.44	$C_{33}H_{41}O_{20}$	$[M]^+$	757.2164	757.2186	-2.91	287.0539$[M-Glu*2-Coumaroyl]^+$, 449.1601$[M-Glu- Coumaroyl]^+$	[3]
8	Delphinidin-3-O-galactoside	57.48	$C_{21}H_{21}O_{12}$	$[M]^+$	465.1025	465.1027	-0.43	303.0487$[M-Gal]^+$, 447.3457$[M-H_2O]^+$	[2]
9	Cyanidin-3-O-(trans-7″-O-coumaroyl)-diglucoside	58.26	$C_{33}H_{41}O_{20}$	$[M]^+$	757.2168	757.2186	-2.38	287.0539$[M-Glu*2-Coumaroyl]^+$, 449.1601$[M-Glu- Coumaroyl]^+$, 611.1591$[M-Coumaroyl]^+$	[3]
10	Cyanidin-3-O-galactoside	58.27	$C_{21}H_{22}O_{11}$	$[M]^+$	449.1087	449.1084	0.67	125.0244$[M-H-C_{15}H_{16}O_8]^-$, 269.0448$[M-H-Gal -H_2O]^-$, 287.0557$[M-H-Gal]^-$	[2]
11	Cyanidin-3-O-glucoside	58.34	$C_{21}H_{22}O_{11}$	$[M]^+$	449.1087	449.1084	0.67	125.0244$[M-H-C_{15}H_{16}O_8]^-$, 269.0448$[M-H-Glu-H_2O]^-$, 287.0557$[M-H-Glu]^-$	Std
12	Cyanidin-3-[2″-(6‴-coumaroyl)-glucosyl]-glucoside	60.14	$C_{36}H_{37}O_{18}$	$[M]^+$	757.1974	757.1974	0.00	287.0539$[M-Glu*2-Coumaroyl]^+$	[2]
13	Pelargonidin-3-glucoside	65.17	$C_{21}H_{21}O_{10}$	$[M]^+$	433.1133	433.1129	1.25	271.0596$[M-Glu]^+$	Std
14	Pelargonidin-3-O-(6″-O-coumaroyl)-diglucoside	67.00	$C_{36}H_{37}O_{17}$	$[M]^+$	741.2027	741.2025	0.27	271.059$[M-Glu*2-Coumaroyl]^+$	[3]
15	Peonidin-3-O-(6″-O-coumaroyl)-glucoside, 5-O-glucoside	67.17	$C_{34}H_{43}O_{20}$	$[M]^+$	771.2333	771.2333	0.00	317.0641$[M-Glu*2-Coumaroyl]^+$, 463.1220$[M-Glu- Coumaroyl]^+$	[3]
16	Delphinidin-3-glucoside	67.93	$C_{21}H_{21}O_{12}$	$[M]^+$	465.1036	465.1028	1.72	303.0495$[M-Glu]^+$	Std

17	Delphinidin-3-O-(6″-O-coumaroyl)-glucoside	67.97	$C_{27}H_{31}O_{16}$	[M]$^+$	611.1601	611.1607	-0.98	303.0487[M-Glu-Coumaroyl]$^+$	[1]
18	Delphinidin	58.24	$C_{15}H_{11}O_7$	[M]$^+$	303.0504	303.0499	1.65	285.0381[M-H_2O]$^+$, 153.0177[M-$C_7H_5O_3$]$^+$, 137.0230[M-$C_8H_9O_3$]$^+$	Std
19	Petunidin-3-O-(6″-O-coumaroyl)-glucoside	83.65	$C_{28}H_{33}O_{16}$	[M]$^+$	625.1762	625.1763	-0.16	317.0642[M-Glu-Coumaroyl]$^+$	[1]
20	Petunidin	83.69	$C_{16}H_{13}O_7$	[M]$^+$	317.0660	317.0656	1.26	302.0418[M-CH_3]$^+$, 274.0468[M-H_2O-OCH_3]$^+$	Std
21	Peonidin-3-O-(6″-O-coumaroyl)-glucoside	87.38	$C_{28}H_{33}O_{15}$	[M]$^+$	609.1821	609.1814	1.15	301.0694[M-Glu-Coumaroyl]$^+$, 461.1213[M- Coumaroyl]$^+$	[3]
22	Peonidin-3-O-glucoside	88.99	$C_{22}H_{23}O_{11}$	[M]$^+$	463.1238	463.1235	0.07	301.0702[M-Glu]$^+$	[2]
					Flavonoid				
23	Catechin-hexoside	4.14	$C_{21}H_{24}O_{11}$	[M-H]$^-$	451.1246	451.1246	0.00	289.0715[M-H-Glu]$^-$, 245.0795[M-H-Glu-COO]$^-$	[2]
24	Catechin	40.46	$C_{15}H_{14}O_6$	[M-H]$^-$	289.0715	289.0712	1.04	182.0281[M-H-$C_6H_5O_2$]$^-$	Std
25	Epicatechin-hexoside	40.64	$C_{21}H_{24}O_1$	[M-H]$^-$	451.1246	451.1246	0.00	289.0715[M-H-Glu]$^-$, 245.0795[M-H-Glu-COO]$^-$	[2]
26	Epicatechin	48.54	$C_{15}H_{14}O_6$	[M-H]$^-$	289.0718	289.0712	2.08	245.0815[M-H-CH_3CHO]$^-$	Std
27	Vicenin II	50.51	$C_{27}H_{30}O_{15}$	[M-H]$^-$	593.1514	593.1512	0.34	285.0397[M-H-Rha-Glu]$^-$, 255.0303[M-H-Glu-Rha-2OH]$^-$, 227.0348[M-H-Glu-Rha-OH-COO]$^-$	[5]
28	Kaempferol-3-O-rhamnosyl-7-O-glucoside	58.29	$C_{33}H_{40}O_{20}$	[M-H]$^-$	755.2047	755.2035	1.59	609.1432[M-H-Rha]$^-$, 446.0828[M-H-Rha-Glu]$^-$, 299.0196[M-H-Rha*2-Glu]$^-$	Database
29	Homoorientin	58.43	$C_{21}H_{20}O_{11}$	[M-H]$^-$	447.0933	447.0931	0.45	285.0399[M-H-Glu]$^-$	Std
30	Vitexin	65.18	$C_{21}H_{20}O_{10}$	[M-H]$^-$	431.0984	431.0985	-0.23	413.0892[M-H-H_2O]$^-$, 311.0555[M-H-$C_4H_8O_4$]$^-$	Std
31	Isovitexin	67.67	$C_{21}H_{20}O_{10}$	[M-H]$^-$	431.0984	431.0985	-0.23	311.0558[M-H-$C_4H_8O_4$]$^-$	Std
32	Rutin	67.98	$C_{27}H_{30}O_{16}$	[M-H]$^-$	609.1458	609.1456	0.33	301.0714[M-H-$C_{12}H_{21}O_9$]$^-$, 300.0270[M-H-$C_{12}H_{22}O_9$]$^-$, 271.0241[M-H-$C_{12}H_{18}O_{11}$]$^-$	Std
33	Kaempferol-3-O-rutinoside	67.98	$C_{27}H_{30}O_{15}$	[M-H]$^-$	593.1514	593.1512	0.34	285.0400[M-H-Glu-Rha]$^-$, 255.0297[M-H-Glu-Rha-2OH]$^-$	[6]
34	Isoquercitrin	71.35	$C_{21}H_{20}O_{12}$	[M-H]$^-$	463.0882	463.0885	-0.65	301.0350[M-H-Glu]$^-$, 248.8878[M-H-Glu-H_2O*3]$^-$	Std
35	Quercetin-3-O-galactoside	71.35	$C_{21}H_{20}O_{12}$	[M-H]$^-$	463.0882	463.0882	0.00	300.0270[M-H-Gal]$^-$, 271.0245[M-H-Gal-2OH]$^-$, 255.0293[M-H-Gal-3OH]$^-$	[2]
36	Dalpanin	72.78	$C_{26}H_{30}O_{12}$	[M-H]$^-$	533.1667	533.1659	1.50	371.1140[M-H-Glu]$^-$, 353.1025[M-H-Glu-H_2O]$^-$, 341.1019[M-H-Glu-CH_2O]$^-$	Database
37	Scolymoside	75.01	$C_{27}H_{30}O_{15}$	[M-H]$^-$	593.1506	593.1507	-0.17	285.0400[M-H-Glu-Rha]$^-$, 255.0297[M-H-Glu-Rha-2OH]$^-$	Database
38	Kaempferol-7-O-neohesperidoside	80.78	$C_{27}H_{30}O_{15}$	[M-H]$^-$	593.1514	593.1512	0.34	285.0402[M-H-Glu-Rha]$^-$, 255.0298[M-H-Glu-Rha-OH*2]$^-$, 227.0354[M-H-Glu-OH*2-CO]$^-$	[6]
39	Isorhamnetin 3-O-neohesperidoside	83.71	$C_{28}H_{32}O_{16}$	[M-H]$^-$	623.1622	623.1612	1.60	315.0507[M-H-Rha-Glu]$^-$, 299.0195[M-H-Rha-Glu-O]$^-$, 271.0244[M-H-Rha-Glu-COO]$^-$, 153.0037[M-H-$C_{21}H_{26}O_{12}$]$^-$	[5]
40	Quercitrin	84.30	$C_{21}H_{20}O_{11}$	[M-H]$^-$	447.0933	447.0931	1.34	284.0322[M-H-Glu]$^-$, 255.0295[M-H-Glu-OH*2]$^-$, 125.0242[M-H-Glu-$C_8H_6O_4$]$^-$	Database
41	Isorhamnetin-3-O-galactoside	86.88	$C_{22}H_{22}O_{12}$	[M-H]$^-$	477.1036	477.1038	-0.42	299.0185[M-Gal]$^-$, 271.024[M-Gal-OCH_3]$^-$	[2]
42	Diosmin	87.36	$C_{28}H_{32}O_{15}$	[M-H]$^-$	607.1670	607.1663	1.15	299.0580[M-H-Rha-Glu]$^-$, 284.0319[M-H-Rha-Glu-CH_3]$^-$	Std
43	Hesperidin	87.38	$C_{28}H_{34}O_{15}$	[M+H]$^+$	609.1821	609.1819	0.34	301.0712[M+H-$C_{12}H_{20}O_6$]$^+$, 125.0241[M-H-$C_{22}H_{28}O_9$]$^-$	Std
44	Isorhamnetin-3-O-glucoside	89.63	$C_{22}H_{22}O_{12}$	[M-H]$^-$	477.1039	477.1038	1.26	299.0185[M-H-Glu]$^-$, 271.0240[M-H-Glu-CO]$^-$	[2]

45	Tectorigenin	97.99	$C_{16}H_{12}O_6$	$[M-H]^-$	299.0556	299.0555	0.33	284.0324[M-H-CH_3]$^-$, 256.0371[M-H-C_2H_5O]$^-$, 227.0348[M-H-$C_3H_5O_2$]$^-$	Database
46	Laricitrin-3-O-rhamnoside	98.74	$C_{22}H_{22}O_{12}$	$[M-H]^-$	477.1039	477.1038	0.21	299.0185[M-Glu]$^-$, 271.024[M-Glu-CO]$^-$	[2]
47	Quercetin	99.58	$C_{15}H_{10}O_7$	$[M-H]^-$	301.0351	301.0348	1.49	179.9983[M-H-$C_6H_2O_3$]$^-$, 153.0033[M-H-$C_8H_4O_3$]$^-$	Std
48	7, 3', 4'-O-methylluteolin	101.61	$C_{18}H_{16}O_6$	$[M-H]^-$	327.2175	327.2171	1.22	190.9284[M-H-$C_8H_{10}O_2$]$^-$, 146.9686[M-H-$C_9H_{13}O_4$]$^-$	Mzcloud
49	Luteolin	102.12	$C_{15}H_{10}O_6$	$[M-H]^-$	285.0402	285.0399	0.99	255.0302[M-H-HCHO]$^-$, 151.0034[M-H-$C_8H_6O_2$]$^-$	Std
50	Kaempferol	102.12	$C_{15}H_{10}O_6$	$[M-H]^-$	285.0399	285.0398	0.35	241.0491[M-H-HCOOH]$^-$, 165.0184[M-H-$C_7H_6O_2$]$^-$, 133.0282[M-H-$C_7H_6O_4$]$^-$, 111.0079[M-H-$C_9H_4O_4$]$^-$	Std
51	Diosmetin	102.36	$C_{16}H_{12}O_7$	$[M-H]^-$	299.0556	299.0555	0.33	284.0324[M-H-CH_3]$^-$, 256.0371[M-H-C_2H_5O]$^-$, 227.0348[M-H-$C_3H_5O_3$]$^-$	Std
52	Querceitn 3-O-(2G-rhamnosylrutinoside)	63.25	$C_{33}H_{40}O_{19}$	$[M-H]^-$	739.2086	739.2086	0.00	285.0390[M-H-Glu-Rha*2]$^-$, 255.0292[M-H-Glu-Rha*2-2OH]$^-$, 593.1500[M-H-Rha]$^-$	[7]
				Phenolic acids					
53	Protocatechuate	21.81	$C_7H_6O_4$	$[M-H]^-$	153.0191	153.0188	1.96	109.0292[M-H-COO]$^-$	Std
54	2, 4-Dihydroxybenzoic acid	27.87	$C_7H_6O_4$	$[M-H]^-$	153.0191	153.0188	1.96	109.0292[M-H-COO]$^-$	Std
55	Panillic acid-hexoside	28.21	$C_{14}H_{18}O_9$	$[M-H]^-$	329.0873	329.0873	0	191.0346[M-H-$C_4H_{10}O_5$]$^-$, 167.0349[M-H-Glu]$^-$, 123.0452[M-H-Glu-COO]$^-$	Database
56	Protocatechuic acid-hexoside	28.32	$C_{13}H_{16}O_9$	$[M-H]^-$	315.072	315.0716	1.27	152.0116[M-H-Glu]$^-$, 108.0217[M-H-Glu-COO]$^-$	Database
57	Neochlorogenic acid	31.81	$C_{16}H_{18}O_9$	$[M-H]^-$	353.0884	353.0873	3.12	191.0561[M-H-Glu]$^-$, 179.0350[M-H-$C_7H_{10}O_5$]$^-$, 135.0451[M-H-$C_9H_{14}O_6$]$^-$	[5]
58	Caffeic acid hexoside/isomer	33.83	$C_{15}H_{18}O_9$	$[M-H]^-$	341.088	341.0878	0.59	179.0350[M-H-Glu]$^-$, 135.0452[M-H-Glu-COO]$^-$	[2]
59	Primeverin/isomer	35.17	$C_{20}H_{28}O_{13}$	$[M-H]^-$	475.1458	475.1452	1.26	385.2652[M-H-$COOCH_3$-OCH_3]$^-$, 311.1040[M-H-Rha]$^-$, 169.0505[M-H-Rha-Glu]$^-$	Database
60	Ferulic acid hexoside/isomer	39.46	$C_{16}H_{20}O_9$	$[M-H]^-$	355.1036	355.1035	0.28	193.0503[M-H-Glu]$^-$, 178.0273[M-H-Glu-CH_3]$^-$, 134.0373[M-H-Glu-CH_3-COO]$^-$	[2]
61	Primeverin/isomer	39.46	$C_{20}H_{28}O_{13}$	$[M-H]^-$	475.1458	475.1452	1.26	385.2652[M-H-$COOCH_3$-OCH_3]$^-$, 311.1040[M-H-Rha]$^-$, 169.0505[M-H-Rha-Glu]$^-$	Database
62	Apigenin-arabinoside I	40.15	$C_{19}H_{28}O_{12}$	$[M-H]^-$	447.1507	447.1503	0.89	284.0331[M-H-Glu]$^-$, 270.0945[M-H-Glu-CH_3]$^-$, 92.6366[M-H-Glu*2-CH_3]$^-$	Database
63	Caffeic acid hexoside/isomer	40.43	$C_{15}H_{18}O_9$	$[M-H]^-$	341.088	341.0878	0.59	179.0350[M-H-Glu]$^-$, 135.0452[M-H-Glu-COO]$^-$	[2]
64	Coumaric acid hexoside/isomer	40.60	$C_{15}H_{18}O_8$	$[M-H]^-$	325.0929	325.0923	1.85	163.0398[M-H-Glu]$^-$, 119.0503[M-H-Glu-COO]$^-$	[2]
65	p-Coumaric acid	40.60	$C_9H_8O_3$	$[M-H]^-$	163.0398	163.0395	1.84	119.0499[M-H-COO]$^-$	Std
66	Chlorogenic acid	42.19	$C_{16}H_{18}O_9$	$[M-H]^-$	353.0874	353.0873	0.28	191.0558[M-H-$C_9H_6O_3$]$^-$	Std
67	Sinapaldehyde	43.00	$C_{11}H_{12}O_4$	$[M-H]^-$	207.0657	207.0656	0.48	177.0546[M-H-CH_4O]$^-$, 149.0599[M-H-CH_4O-CO]$^-$	Database
68	Cryptochlorogenic acid	43.13	$C_{16}H_{18}O_9$	$[M-H]^-$	353.0882	353.0873	2.55	191.0561[M-H-Glu]$^-$, 179.0350[M-H-$C_7H_{10}O_5$]$^-$, 135.0451[M-H-$C_9H_{14}O_6$]$^-$	[5]
69	Glucosyl-6-O-glucosyl-4-hydroxycinnamic acid	44.81	$C_{21}H_{28}O_{13}$	$[M-H]^-$	487.1454	487.1452	0.41	145.0296[M-H-Glu-Rha-H_2O]$^-$, 367.1032[M-H-$C_7H_6O_2$]$^-$, 325.0924[M-H-Rha]$^-$, 163.0400[M-H-Glu-Rha]$^-$	[7]
70	Coumaric acid hexoside/isomer	44.84	$C_{15}H_{18}O_8$	$[M-H]^-$	325.0929	325.0929	0	163.0398[M-Glu]$^-$, 119.0503[M-Glu-COO]$^-$	[2]

71	Ferulic acid	45.39	$C_{10}H_{10}O_4$	$[M-H]^-$	193.0503	193.0501	1.04	178.0271$[M-H-CH_3]^-$, 134.0371$[M-H-C_2H_3O_2]^-$	Std
72	Ferulic acid hexoside/isomer	45.39	$C_{16}H_{20}O_9$	$[M-H]^-$	355.1036	355.1035	0.28	193.0503$[M-H-Glu]^-$, 178.0273$[M-H-Glu-CH_3]^-$, 134.0373$[M-H-Glu-CH_3-COO]^-$	[2]
73	Phloridzin	46.19	$C_{21}H_{24}O_{10}$	$[M-H]^-$	435.1296	435.1297	-0.23	315.0872$[M-H-C_8H_7O]^-$, 273.0760$[M-H-Glu]^-$, 167.0349$[M-H-C_{13}H_{15}O_6]^-$	[2]
74	Dimethoxy-Glucopyranosyl-cinnamic acid	48.54	$C_{17}H_{22}O_{10}$	$[M-H]^-$	385.1143	385.1135	2.08	325.0941$[M-H-CH_2-HCOOH]^-$, 163.3988$[M-H-C_2H_4O_2-Glu]^-$	Database
75	Protocatechuic acid	51.71	$C_7H_6O_4$	$[M-H]^-$	153.0191	153.0188	1.96	109.0292$[M-H-COO]^-$	[8]
76	Psoralidin	101.27	$C_{20}H_{16}O_5$	$[M-H]^-$	335.0925	335.0922	0.9	-	Std
77	6-Gingerol	106.20	$C_{17}H_{26}O_4$	$[M-H]^-$	293.1758	293.1753	1.71	236.1054$[M-H-C_4H_9]^-$, 221.1545$[M-H-C_5H_{12}]^-$	Std
				Organic acid					
78	2-Ketobutyric acid	3.79	$C_4H_6O_3$	$[M-H]^-$	101.0239	101.0239	0.00	83.6712$[M-H-H_2O]^-$, 73.0294$[M-H-C_2H_4]^-$, 55.0188$[M-H-HCOOH]^-$	Database
79	Quinic acid	4.41	$C_7H_{12}O_6$	$[M-H]^-$	191.0556	191.0556	0.00	174.05$[M-H-H_2O]^-$, 135.0298$[M-H-COO]^-$, 86.04$[M-H-H_2O-C_3H_4O_3]^-$	[9]
80	Citric acid	9.08	$C_6H_8O_7$	$[M-H]^-$	191.0196	191.0192	2.09	173.0090$[M-H-H_2O]^-$, 129.0195, $[M-H-C_2H_6O_4]^-$, 117.0192$[M-H-C_2H_5O_3]^-$	[9]
81	Tetradecanoic acid	115.55	$C_{14}H_{28}O_2$	$[M-H]^-$	227.2011	227.2011	0.00	183.0272$[M-H-HCOOH]^-$, 155.1173$[M-H-HCOOH-C_2H_4]^-$, 84.0814$[M-H-HCOOH-C_7H_{15}]^-$	Std
82	Palmitoleic acid	115.99	$C_{16}H_{30}O_2$	$[M-H]^-$	253.2168	253.2167	0.39	-	Std
83	Linoleic Acid	116.13	$C_{18}H_{32}O_2$	[M-H]-	279.2324	279.2325	-0.36	-	Std
84	Palmitic acid	118.77	$C_{16}H_{32}O_2$	$[M-H]^-$	255.2324	255.2321	1.18	-	Std
85	Oleic acid	118.85	$C_{18}H_{34}O_2$	$[M-H]^-$	281.2481	281.2482	-0.36	-	Std
86	Isooctadecanoic acid	123.42	$C_{18}H_{36}O_2$	$[M-H]^-$	283.2637	283.2637	0.00	-	Std
				Amino acid					
87	Histidine	3.53	$C_6H_9N_3O_2$	$[M+H]^+$	156.0773	156.0773	0.00	137.0356$[M+H-NH_3]^+$, 67.0300$[M+H-C_3H_5O_2N]^+$	Mzcloud
88	Serine	3.55	$C_3H_7NO_3$	$[M+H]^+$	106.0504	106.0503	0.94	88.0390$[M+H-H_2O]^+$, 60.0445$[M+H-HCOOH]^+$	Mzcloud
89	Glutamic acid	3.85	$C_5H_9NO_4$	$[M+H]^+$	146.0453	146.0453	-0.68	128.0353$[M+H-H_2O]^+$, 102.0546$[M+H-COO]^+$, 87.0087$[M+H-NH_3-CH_2O_2]^+$	Mzcloud
90	2-Aminoisobutyric acid	3.87	$C_4H_9NO_2$	$[M+H]^+$	104.0712	104.0711	0.96	87.0438$[M+H-NH_3]^+$, 60.0809$[M+H-COO]^+$	Mzcloud
91	Asparagine	3.96	$C_4H_8N_2O_3$	$[M+H]^+$	133.0613	133.0613	0.00	116.0346$[M+H-NH_3]^+$, 88.0305$[M+H-NH_3-CO]^+$, 87.0269$[M+H-HCOOH]^+$, 70.0659$[M+H-HCOOH-NH_3]^+$	Mzcloud
92	Allysine	4.13	$C_6H_{11}NO_3$	$[M+H]^+$	146.0817	146.0817	0.00	100.0762$[M+H-HCOOH]^+$, 128.0708$[M+H-CO]^+$	Mzcloud
93	Proline	4.42	$C_5H_9NO_2$	$[M+H]^+$	116.0712	116.0711	0.86	70.0660$[M+H-HCOOH]^+$, 55.0552$[M+H-HCOOH-CH_3]^+$	Mzcloud
94	Valine	6.48	$C_5H_{11}NO_2$	$[M+H]^+$	118.0868	118.0867	0.85	76.9338$[M+H-C_3H_6]^+$, 58.0661$[M+H-C_3H_8-H_2O]^+$	Mzcloud
95	Pipecolic acid	6.51	$C_6H_{11}NO_2$	$[M+H]^+$	130.0868	130.0868	0.00	84.0815$[M+H-HCOOH]^+$, 56.0505$[M+H-C_2H_4NO_2]^+$	Mzcloud
96	Tyrosine	16.98	$C_9H_{11}NO_3$	$[M+H]^+$	182.0817	182.0816	0.55	141.9587$[M+H-COOH]^+$, 113.9640$[M+H-COOH-CO]^+$	Mzcloud
97	Phenylalanine	25.03	$C_{15}H_{21}NO_7$	$[M+H]^+$	328.1382	328.1396	-4.27	310.1272$[M+H-H_2O]^+$, 292.1168$[M+H-2H_2O]^+$, 166.0718$[M+H-Glu]^+$	[7]
98	Tryptophan	35.09	$C_{11}H_{12}N_2O_2$	$[M+H]^+$	205.0967	205.0977	-4.88	188.1428$[M+H-OH]^+$, 159.0911$[M+H-COO-NH_2]^+$, 146.0595$[M+H-C_2H_3O_2]^+$	[7]

99	N-malonyl-tryptophan	65.17	$C_{14}H_{14}N_2O_5$	$[M+H]^+$	291.0971	291.0981	-3.44	245.0910$[M+H-HCOOH]^+$, 209.0700$[M+H-C_4H_7O_2]^+$, 130.0647$[M+H-C_9H_7NO_2]^+$	[6]
					Saccharides				
100	Erythrulose	3.79	$C_4H_8O_4$	$[M-H]^-$	119.0344	119.0344	0.00	71.0138$[M-H-CH_4O_2]^-$, 59.0138$[M-H-C_2H_4O_2]^-$	Mzcloud
101	Cellobiose	4.50	$C_{12}H_{22}O_{11}$	$[M-H]^-$	341.1085	341.1084	0.29	179.0350$[M-H-Glu]^-$, 135.0352$[M-H-COO]^-$	Mzcloud
102	Sedoheptulose	4.90	$C_7H_{14}O_7$	$[M-H]^-$	209.0661	209.0661	0.00	165.0764$[M-H-CH_3O_2]^-$	Mzcloud
103	Thyminose	8.91	$C_5H_{10}O_4$	$[M-H]^-$	133.0501	133.0501	0.00	115.0036$[M-H-H_2O]^-$, 87.0087$[M-H-H_2O -CO]^-$, 71.0138$[M-H-H_2O -C_2H_4O]^-$	Mzcloud

Std：Compounds confirmed by standards;

Database：Verified through self built database matching;

Mzcloud：Identification of secondary fragment matching through Mzcloud database;

Table S2. Some of key metabolites differentially accumulated between LNS vs HNS and LNT vs HNT.

**Name**	**LNS vs.HNS**			**LNT vs. HNT**		
	**^{a}log$_2$FC**	**^{b}*P***	**VIP>1**	**log$_2$FC**	***P***	**VIP>1**
Asparagine				-0.99	*	1.13
Serine				1.04	*	1.07
Valine	1.90	**	1.17	1.21	*	1.66
Tyrosine	1.65	*	1.12	1.34	*	1.00
Rutin	-1.25	**	1.67	-1.77	***	1.44
Quercetin	-1.56	**	2.06	1.36	**	1.25
Epicatechin	-3.22	**	1.22			
Kaempferol-3-O-rutinoside	-4.18	*	1.58	-3.22	*	1.36
hydroxy- trimethoxy flavone	-5.74	***	1.83	-3.39	**	1.48
Cyanidin-3-O-diGlucoside	-3.25	***	2.01	-1.60	***	1.32
Cyanidin-glucuronide	-1.53	***	2.16	-1.16	***	1.01
Cyanidin-3-O-(6"-O-coumaroyl)-Glucoside/isomer	-4.49	***	1.72	-3.41	**	1.32
Cyanidin-3-O-(6"-O-coumaroyl)-Glucoside/isomer	-1.93	*	1.02	-1.43	*	1.15
Cyanidin-3-O-(trans-7"-O-coumaroyl)-diGlucoside	-7.09	*	2.18	-8.65	*	1.84
Petunidin	-0.25	***	1.58	-0.64	**	1.02
Petunidin 3-O-rutinoside-glucose	2.62	**	1.40			
Peonidin-3-O-(6"-O-coumaroyl)-Glucoside, 5-O-Glucoside	-5.82	***	2.12	-2.48	*	1.15
Petunidin-3-O-(6"-O-coumaroyl)-Glucoside	-0.22	***	1.77	-0.70	***	1.59
Delphinidin	-1.47	***	2.00	-1.27	***	1.29
Delphinidin-3-O-galactosideb	-1.54	***	1.34	-1.14	***	1.17
Delphinidin-glucuronide	-4.23	***	1.75	-5.77	***	1.89
Delphinidin-dihexoside (II)a	-3.64	***	1.44	-3.61	***	1.54
Kaempferol-3-O-rhamnosylgalactoside-7-O-glucoside	-5.85	***	2.16	-2.58	***	1.39
Pelargonidin-3-Glu/isomer	-3.74	***	1.40	-2.77	***	1.33
p-Coumaric acid	-2.93	***	1.37	-2.91	**	1.26
Chlorogenic acid	-4.18	***	1.46	-4.71	**	1.57
protocatechuic acid- hexoside	1.94	***	1.38	3.75	***	1.57

Sinapaldehyde	-3.81	***	1.63	-4.60	***	1.70
Oleic acid				-2.54	***	1.28
Linoleic Acid				-4.06	***	1.63
Palmitoleic acid	2.18	*	1.02	-1.70	*	1.25
2-Ketobutyric acid	1.41	*	1.56	0.35	***	1.07
3-Isopropylmalate	2.58	***	1.48	1.83	***	1.21
Sinapaldehyde	-3.81	***	1.63	-4.60	***	1.70
Apigenin-arabinoside I				1.91	***	1.17

Table S3. The number of clean reads per library ranged from 42,900,204 to 611,70,750.

Item	Genes number	GC percentage	N50 percentage	N50 length	Max length	Min length	Average length
Value	65398	43.61%	17.12%	1535 bp	15681 bp	201 bp	874 bp

Table S4. Gene annotation in four databases.

Annotated Database	Number of unigenes	Percentage
All	65398	100.00%
Annotation	38642	59.09%
KEGG	31405	48.02%
COG	24217	37.03%
SwissProt	28187	43.10%
Nr	36143	55.27%

[1] Tian Z, Aierken A, Pang H, et.al. Constituent analysis and quality control of anthocyanin constituents of dried Lycium ruthenicum Murray fruits by HPLC–MS and HPLC–DAD [J]. J Liq Chromatogr Relat Technol, 2016, 39(9): 453-458.

[2] Ancillotti C, Ciofi L, Rossini D, et.al. Liquid chromatographic/electrospray ionization quadrupole/time of flight tandem mass spectrometric study of polyphenolic composition of different Vaccinium berry species and their comparative evaluation [J]. Anal Bioanal Chem, 2017, 409(5): 1347-1368.

[3] Zhang M, Ma J, Bi H, et.al. Characterization and cardioprotective activity of anthocyanins from Nitraria tangutorum Bobr. by-products [J]. Food & function, 2017, 8(8): 2771-2782.

[4] Jin H, Zhao J, Zhou W, et.al. Preparative separation of a challenging anthocyanin from Lycium ruthenicum Murr. by two-dimensional reversed-phase liquid chromatography/hydrophilic interaction chromatography [J]. RSC advances, 2015, 5(76): 62134-62141.

[5] Wu T, Lv H, Wang F, et.al. Characterization of Polyphenols from Lycium ruthenicum Fruit by UPLC-Q-TOF/MS(E) and Their Antioxidant Activity in Caco-2 Cells [J]. J Agric Food Chem, 2016, 64(11): 2280-2288.

[6] Song Q, Xia X, Ji C, et.al. Optimized flash extraction and UPLC-MS analysis on antioxidant compositions of Nitraria sibirica fruit [J]. J Pharm Biomed Anal, 2019, 172: 379-387.

[7] Zhao J Q, Wang Y M, Yang Y L, et.al. Isolation and identification of antioxidant and α-glucosidase inhibitory compounds from fruit juice of Nitraria tangutorum [J]. Food Chem, 2017, 227: 93-101.

[8] Zhao J, Xu F, Ji T, et.al. A New Spermidine from the Fruits of Lycium ruthenicum [J]. Chem Nat Compd, 2014, 50: 880-883.

[9] Turghun C, Bakri M, Abdulla R, et.al. Comprehensive characterisation of phenolics from Nitraria sibirica leaf extracts by UHPLC-quadrupole-orbitrap- MS and evaluation of their anti-hypertensive activity [J]. J Ethnopharmacol, 2020, 261: 113019.
